# Investigation on Potential ESKAPE Surrogates for 222 and 254 nm Irradiation Experiments

**DOI:** 10.3389/fmicb.2022.942708

**Published:** 2022-07-01

**Authors:** Anna-Maria Gierke, Martin Hessling

**Affiliations:** Institute of Medical Engineering and Mechatronics, Ulm University of Applied Sciences, Ulm, Germany

**Keywords:** disinfection, ESKAPE pathogen, surrogates, far-UVC, photoinactivation, radiation, UVC

## Abstract

**Background:**

Due to the increase in multidrug-resistant pathogens, it is important to investigate further antimicrobial options. In order not to have to work directly with pathogens, the investigation of possible surrogates is an important aspect. It is examined how suitable possible surrogate candidates for ESKAPE pathogens are for UVC applications. In addition, the inactivation sensitivities to 222 and 254 nm radiation are compared in relation.

**Methods:**

Non-pathogenic members (*Enterococcus mundtii, Staphylococcus carnosus, Acinetobacter kookii, Pseudomonas fluorescens* and *Escherichia coli*) of genera of ESKAPE strains were photoinactivated in PBS with irradiation wavelengths of 222 and 254 nm (no non-pathogenic *Klebsiella* was available). Log reduction doses were determined and compared to published photoinactivation results on ESKAPE pathogens. It was assumed that non-pathogenic bacteria could be designated as surrogates for one wavelength and one ESKAPE strain, if the doses were between the 25 and 75% quantiles of published log reduction dose of the corresponding pathogen.

**Results:**

For all non-pathogen relatives (except *A. kookii*), higher average log reduction doses were required for irradiation at 222 nm than at 254 nm. Comparison by boxplot revealed that five of eight determined log reduction doses of the possible surrogates were within the 25 and 75% quantiles of the data for ESKAPE pathogens. The measured log reduction dose for non-pathogenic *E. coli* was above the 75% quantile at 222 nm, and the log reduction dose for *S. carnosus* was below the 25% quantile at 254 nm.

**Conclusion:**

For more than half of the studied cases, the examined ESKAPE relatives in this study can be applied as surrogates for ESKAPE pathogens. Because of lack of data, no clear statement could be made for *Enterococcus faecalis* at 222 nm and *Acinetobacter baumannii* at both wavelengths.

## Introduction

In hospitals and care facilities, nosocomial infections can be spread through various causes, such as direct and indirect contact between patients and doctors, respectively, nurses. These infections are caused by various microorganisms such as bacteria, viruses, fungi and parasites (Santajit et al., 2016). The proportion of antimicrobial-resistant pathogens (AMR) has increased over the years. More than 33,000 deaths and 874,000 disability-adjusted life years from AMR infections are recorded in Europe every year. The resulting cost is $1.5 billion (Oliveira et al., 2020). In the United States, over 2 million illnesses are caused by multidrug-resistant organisms each year (Burnham et al., 2019). For this reason, the World Health Organization (WHO) published a list of the development of new antimicrobial resistances in 2017, in which all relevant resistant pathogens are represented. The ESKAPE pathogens (*Enterococcus faecium, Staphylococcus aureus, Klebsiella pneumoniae, Acinetobacter baumannii, Pseudomonas aeruginosa*, and members of *Enterobacterales*) were given high priority (Shrivastava et al., 2018).

One possible antimicrobial measure is the application of ultraviolet (UV) radiation, in particular the utilization of radiation in the germicidal UVC wavelength range of 200–280 nm (Kowalski, 2010). In this regard, there are different UVC studies with LEDs related to ESKAPE pathogens (Mariita and Randive, 2021). Cyclobutane pyrimidine dimers (CPD) and 6–4 photoproducts are formed in deoxyribonucleic acid (DNA) by ultraviolet radiation (Hung et al., 2020). Since this prevents replication and transcription, cytotoxic and mutagenic effects can occur, up to cell death (Harm, 1980).

Additionally, there are many studies on low-pressure mercury lamps emitting at 254 nm for the photoinactivation of various microorganisms. It was determined that the peak emission wavelength is close to the absorption peak of DNA and thus the microorganisms are effectively damaged (Setlow and Doyle, 1957; Harm, 1980; Rahmani et al., 2010). With an excimer lamp emitting at 222 nm, on the other hand, the emission wavelength is not only in the absorption range of DNA, but also in the absorption range of proteins (Setlow and Doyle, 1957; Voet et al., 1963; Sosnin et al., 2005; Taylor et al., 2020). Therefore, both radiation sources represent different methods of photoinactivation.

Low-pressure mercury lamps have been applied for radiation disinfection in the UVC wavelength range for over 100 years. However, this source of radiation is assumed to be harmful to humans, which is why krypton chloride (KrCl) excimer lamps are increasingly being investigated (Sosnin et al., 2005). Due to the much higher absorption by intracellular proteins at its 222 nm peak emission wavelength, a lower health risk in application for humans is hoped for as well as an antimicrobial impact comparable to low-pressure mercury lamps (Hessling et al., 2021b). Furthermore, it should be noted that the top layer of skin protects the deeper skin layers from the radiation due to the dead protein-rich cells of the stratum corneum. However, irradiation also inactivates microorganisms, which are part of the natural skin microbiome. In the long run, this can change the composition of the skin microbiome and may thus have an impact on the cutaneous immune system (Mariita et al., 2022).

Microbiological work with pathogens requires a laboratory with biosafety level of 2 or higher. The more dangerous the microorganism, the heavier the safety requirement. Nevertheless, even in biosafety laboratories, there is always a risk of accidents or infections occurring as happened, for example, for SARS-CoV in 2004 (Normile, 2004; Della-Porta, 2008). Hazard reduction can be helped by microbial surrogates that behave similarly to the pathogen of interest but are less hazardous or, ideally, not pathogenic at all. The latter then allows work to be performed outside of high security labs, which is of great importance since such laboratories are limited in their availability.

The importance of surrogates has become particularly evident during the coronavirus pandemic. Many virus reduction developments were performed or tested using surrogates (Hulkower et al., 2011; Casanova and Waka, 2013; Ahmed et al., 2020; Whitworth et al., 2020; Singh et al., 2021; String et al., 2021; Schirtzinger et al., 2022; Serrano-Aroca, 2022). To our knowledge, for example, not a single air disinfection system in the world has been tested on SARS-CoV-2 in a biosafety lab (Hessling et al., 2021b). Tests have only been conducted with less pathogenic surrogates (Ludwig-Begall et al., 2020; Knaus et al., 2021).

Surrogates are not restricted for virus applications but also used in the context of bacterial pathogens. This typically involves either field tests on the spread of microorganisms (Park et al., 2018; Baker et al., 2021) or the effect of disinfection measures, e.g., in the food sector (Griffiths et al., 1998; Niebuhr et al., 2008; Gurtler et al., 2010; Ingham et al., 2010; Yun et al., 2013; Kopit et al., 2014; Orlowska et al., 2015; Hu and Gurtler, 2017; Liu et al., 2018; Rattanakul and Oguma, 2018; Acuff et al., 2020). In some cases, the use of surrogates in connection with antimicrobial measures or their tests is even prescribed or recommended by authorities (DIS/ISO 16604 (2005-02-25), ASTM F1671-07, DIN EN 14583). For some ESKAPE pathogens, like *S. aureus*, *P. aeruginosa*, and pathogenic *E. coli*, some investigations on suitable surrogates for different applications have been reported (Que et al., 2000; Stutzmann Meier et al., 2001; Yun et al., 2013; Orlowska et al., 2015; Lai et al., 2016; Rattanakul and Oguma, 2018; Acuff et al., 2020). However, only a few of these studies have dealt with UVC disinfection and not all ESKAPE pathogens are covered. Therefore, there is still a need to catch up with regard to suitable surrogates. For example, in a literature review, we found that touch screens in healthcare settings are contaminated with bacteria such as ESKAPE pathogens (Hessling et al., 2021a). Surrogates would now be desirable for the development and testing of suitable disinfection measures or devices, so that these tests could be performed without risk, e.g., at least partially outside the hospital or biosafety laboratory.

Therefore, the aim of this work is to investigate the photoinactivation of non-pathogenic members of genera of ESKAPE pathogens at 222 and 254 nm. On this occasion, statements can be made about possible applications of non-pathogenic ESKAPE relatives as surrogates to the pathogenic ESKAPE strains. This would simplify further investigations into the inactivation of the ESKAPE strains, as they can be carried out outside a BSL-2 laboratory. Furthermore, the results would show independence of inactivation with respect to pathogenicity.

Another aspect of this study is to compare the antimicrobial impact of irradiation at both UVC wavelengths (222 and 254 nm), which differ in DNA and protein absorption and might lead to different photoinactivation properties. As already mentioned, the utilization of irradiation sources with 254 nm emissions are harmful to humans. If radiation at both wavelengths exhibit similar antimicrobial properties, future applications with KrCl excimer lamps would be a safe and user-friendly alternative to low-pressure mercury lamps (Eadie et al., 2021).

## Materials and Methods

For the following experiments, the type strains *Staphylococcus carnosus* (DSM20501), *E. coli* (DSM498), *Acinetobacter kookii* (DSM29071), *Pseudomonas fluorescens* (DSM4358) and *Enterococcus mundtii* (DSM4838) were obtained from DSMZ (Deutsche Sammlung für Mikroorganismen und Zellkulturen, Braunschweig, Germany). The media recommended by DSMZ were used (Supplementary Table 2). All strains with the exception of *P. fluorescens* were cultured at 37°C to the mid-exponential phase and then centrifuged at 7,000*g* for 5 min. *P. fluorescens* was cultivated at 30°C followed by the same process when reaching the mid-exponential phase. The resultant pellet was then resuspended and washed with phosphate buffered saline (PBS) twice. Then, the suspension was diluted to a population density of 3.7 × 10^6^ to 5.0 × 10^7^ colony forming units (CFU)/ml. A transmission of approximately 50% was observed in a 10 mm quartz cuvette at 222 and 254 nm. For the irradiation experiments, a layer thickness of only 3 mm was chosen, to reduce average absorption to about 10% of the incident radiation. The measurement was carried out with a spectrophotometer (SPECORD 250 PLUS double beam spectrophotometer, Analytik Jena, Germany). Due to the safety level of the available laboratory, only experiments with non-pathogenic bacteria were performed. Furthermore, no non-pathogenic relative of the Klebsiella genus was found since, the only known candidate *Klebsiella singaporensis* (formerly risk group 1) was renamed and reclassified to *Klebsiella variicola* (risk group 2).

**Figure 1:**
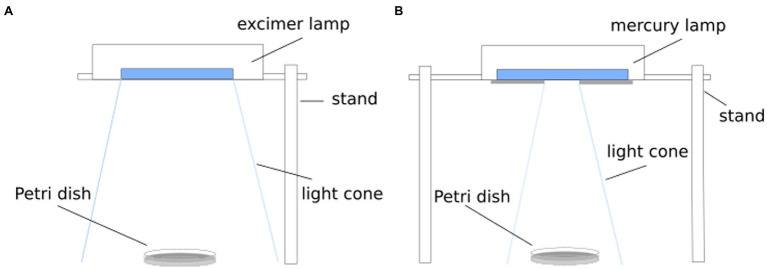
Schematic setup for inactivation experiment. For radiation of 222 nm, a KrCl excimer lamp was applied **(A)** and a mercury vapor lamp was used for a radiation wavelength of 254 nm **(B)**.

In the following investigations, two wavelengths (222 and 254 nm) were applied for irradiation (Spectra are available in Supplementary Figure 1 and Supplementary Table 1). For 222 nm irradiation, a krypton chloride excimer lamp (Ushio Care 222 Modell B1, Ushio Europe B.V., Japan) was placed over the sample (Figure 1A). A mercury vapor lamp (TUV 15 W/G15T8, Philips, Netherlands) was used for the 254 nm experiments, whereby the lamp was partially covered for intensity reduction (Figure 1B). A 3 ml of the bacteria suspension was pipetted in a 55 mm diameter Petri dish and placed in the center under the light source. An intensity of 0.04 mW/cm^2^ was chosen for 222 nm and an intensity of 0.18 mW/cm^2^ for 254 nm. The irradiation intensity was measured in each setup with a UV photometric detector (X1 Optometer, Gigahertz-Optik GmbH, Germany). To reduce the possibility of photoreactivation after irradiation, the taken samples and the streaked plates were covered with aluminum foil.

After an incubation period of 48 h, the grown colonies were counted and converted to colony forming units per ml. The results were presented as a log reduction with respect to the starting concentration. The graphical representations and the generated fit curves were created with Matlab R2021a (MathWorks, Natick, United States of America). All experiments were repeated at least three times in triplicates in different dilutions for each run.

Due to safety issues only investigations on non-pathogenic microorganisms could be carried out in the available laboratory. The radiation and biosafety regulations were also taken into account (Mariita et al., 2022). Therefore, for comparing the experimental results on non-pathogenic bacteria to the ESKAPE pathogen data, a literature research had to be executed. A search was performed for ESKAPE pathogens and for the individual representatives of these pathogens. Compared to *Enterococcus faecalis*, barely any data on photoinactivation were found for *Enterococcus faecium*. Although usually *E. faecium* is counted among the ESKAPE pathogens, some studies also include *E. faecalis* (Nakonieczna et al., 2019; de Macedo et al., 2021; Li et al., 2021). Besides the fact that the two bacteria are closely related, *E. faecalis* is also listed alongside *E. faecium* among the resistant pathogens (Palmer et al., 2012; Murray et al., 2022). One study describes a log reduction dose of 4.5 mJ/cm^2^ for *E. faecium* (Martiny et al., 1988). This dose is close to the determined log reduction doses of *E. faecalis* with 3.67 ± 1.73 mJ/cm^2^, which is why the data of *E. faecalis* instead of *E. faecium* were used in this study.

Furthermore, various criteria for published studies had to be fulfilled before they were included in this analysis, such as the applied type of lamps. Additionally, it had to be an open experimental setup under aerobic conditions and the irradiated medium consisted of PBS, water, salted water or ringer’s solution. An open vessel such as a beaker or a petri dish had to be used for irradiation.

From these studies, the average log reduction doses for wavelengths in the UVC and far-UVC range were determined. For this purpose, average log reduction doses up to log 3 reduction were read from tables or directly from the figures and then, the value for the average log reduction was determined.

It was assumed that if log reduction doses of non-pathogenic surrogate candidate are within the 25 and 75% quantiles of the published results of the ESKAPE pathogen of interest, the candidate is an appropriate surrogate. The graphical representation for this study was created using Origin 2021b (OriginLab Corporation, Northampton, MA, United States of America).

## Results

**Figure 2:**
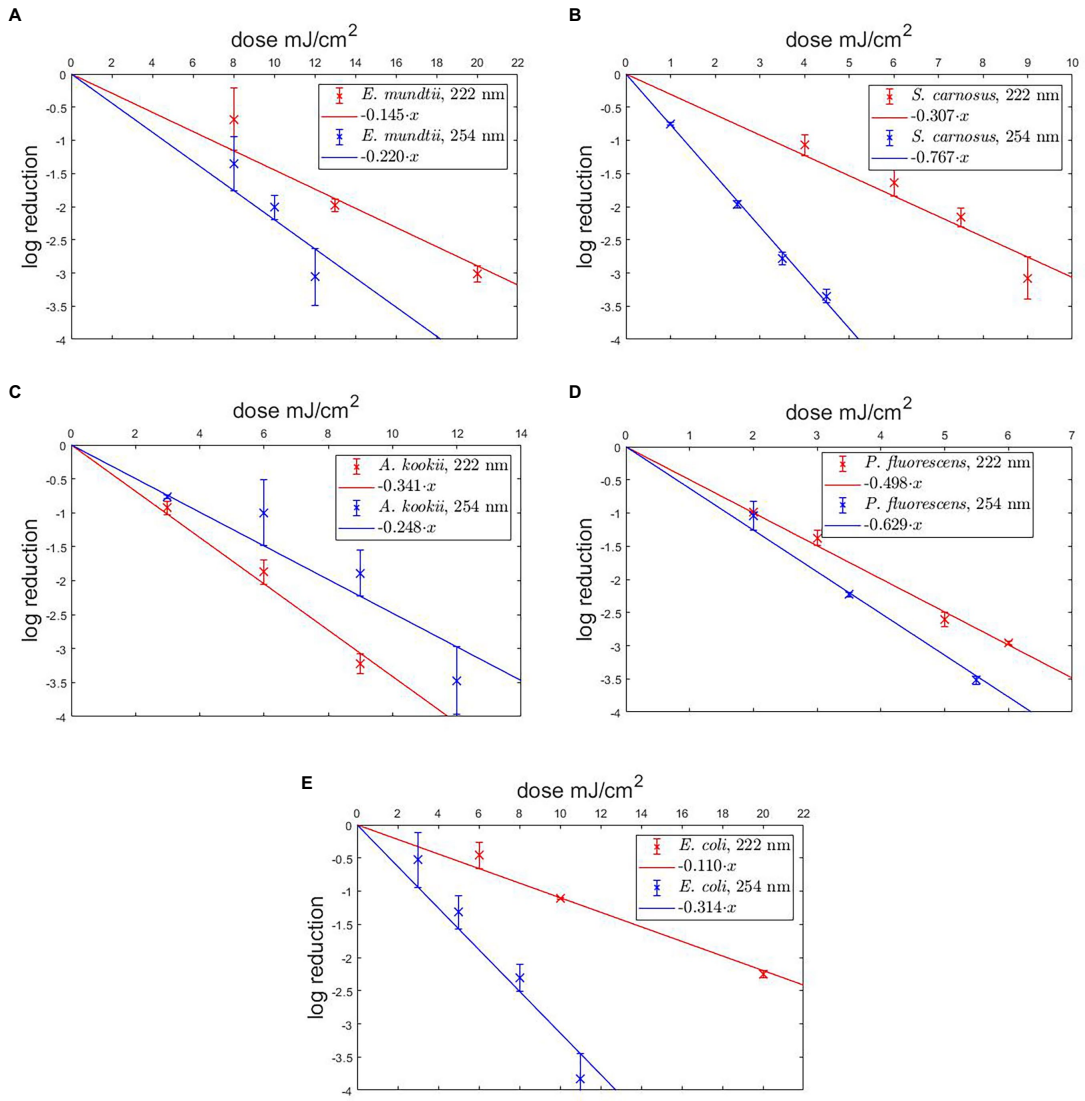
Photoinactivation of non-pathogenic bacteria for 222 nm and 254 nm. The results are presented for *Enterococcus mundtii* **(A)**, *Staphylococcus carnosus* **(B)**, *Acinetobacter kookii* **(C)**, *Pseudomonas fluorescens* **(D)**, *Escherichia coli* **(E)**. A linear fit was added for the determination of the UVC/far-UVC sensitivity.

The aim of this study was to compare photoinactivation using non-pathogenic relatives of the ESKAPE pathogens and to evaluate whether the non-pathogenic relatives were suitable as surrogates. This had been performed for two wavelengths (222 and 254 nm). The linearly fitted results are presented in the half-logarithmic representation in Figure 2 and the irradiation doses are given in Table 1. The data marked in red correspond to the results for 222 nm and the blue marked data correspond to the results for 254 nm irradiation.

**Table 1:** Overview of calculated average log reduction doses for various bacteria and wavelength.

	222 nm average log reduction dose (mJ/cm^2^)	254 nm average log reduction dose (mJ/cm^2^)	Ratio (dose_222nm_/dose_254nm_)
*E. mundtii*	6.90 ± 0.56	4.54 ± 0.34	1.52
*S. carnosus*	3.28 ± 0.50	1.26 ± 0.06	2.60
*A. kookii*	2.93 ± 0.35	4.03 ± 0.74	0.73
*P. fluorescens*	2.01 ± 0.14	1.59 ± 0.12	1.26
*E. coli*	9.10 ± 2.11	3.19 ± 0.89	2.85
			(Average 1.79)

The values are the inverse of the inactivation rate constants of the fitted curves in Figure 2. The errors derive from the standard deviation.The values are the inverse of the inactivation rate constants of the fitted curves in Figure 2. The errors derive from the standard deviation.

The results of the log reduction *via* the irradiation dose were determined with respect to the initial value of the population density. In Figure 2A, the photoinactivation curves of *E. mundtii* at a wavelength of 222 and 254 nm are presented. Here, *E. mundtii* was inactivated on average by a 1.52-fold higher log reduction dose at 222 nm than the determined dose at 254 nm (Table 1). For *S. carnosus*, an average log reduction at a wavelength of 222 nm was obtained with an irradiation dose of 3.28 ± 0.50 mJ/cm^2^ (Figure 2B). For the wavelength of 254 nm, an average irradiation dose of 1.26 ± 0.06 mJ/cm^2^ was observed for a log reduction. For *A. kookii*, an irradiation dose of 4.03 ± 0.74 mJ/cm^2^ at a wavelength of 254 nm was determined in the irradiation experiments for an average log reduction (Figure 2C). Compared to the results for 222 nm, the log reduction dose to inactivate *A. kookii* was below the dose at 254 nm. Figure 2D presents the results of photoinactivation of *P. fluorescens*. From data for the wavelength at 222 nm, it was determined that for a log reduction a dose of 2.01 ± 0.14 mJ/cm^2^ was required. For a wavelength of 254 nm, a 26% lower dose was required for a log reduction. For *S. carnosus* and *P. fluorescens*, a lower reduction dose was sufficient for photoinactivation at a wavelength of 254 nm than at 222 nm. Figure 2E reveals the results of photoinactivation of *E. coli*. For 222 nm, an average log reduction was achieved by an irradiation dose of 9.10 ± 2.11 mJ/cm^2^. Comparing with the irradiation dose for a wavelength of 254 nm*, E. coli* was inactivated with a threefold lower dose than for a wavelength of 222 nm. For *S. carnosus*, *E. coli* and *E. mundtii*, the values were further apart. On average in these experiments, a 1.79-fold higher log reduction dose at 222 nm was needed to reduce the non-pathogenic bacteria related to the ESKAPE pathogens (Table 1).

A list of average log reduction doses of ESKAPE pathogens is represented in Table 2. The wide range of resulting doses by different research groups were described in various review articles and other literature (Malayeri et al., 2016; Hessling et al., 2021b; Masjoudi et al., 2021).

**Table 2:** Overview of median log reduction doses for ESKAPE pathogens retrieved from literature.

	222 nm median log reduction dose (mJ/cm^2^)	254 nm median log reduction dose (mJ/cm^2^)	Ratio (dose_222nm_/dose_254nm_)	Additional information [single average log reduction doses (mJ/cm^2^)]	
				222 nm	254 nm
*E. faecalis*	8.36 ± 1.09	3.67 ± 1.73	2.28	9.13 (Clauss et al., 2009), 7.59 (Nerandzic et al., 2012)	6.03 (Clauss et al., 2009), 7.11 (Chen et al., 2016), 3.67 (Moreno-Andrés et al., 2016), 3.42 (Moreno-Andrés et al., 2017), 3.39 (Wang et al., 2020)
*S. aureus*	3.37 ± 1.48	2.43 ± 0.79	1.39	3.24 (Matafonova et al., 2008), 4.68 (Clauss et al., 2009), 4.60 (Clauss, 2006), 2.19 (Taylor et al., 2020), 4.88 (Nerandzic et al., 2012), 3.51 (Gates, 1930), 1.30 (Kang et al., 2018), 1.31 (Narita et al., 2020),	1.43 (Narita et al., 2020), 2.43 (Clauss et al., 2009), 2.43 (Clauss, 2006), 2.60 (Sharp, 1939), 3.90 (Chang et al., 1985), 1.72 (Kang et al., 2018), 2.71 (Yang et al., 2020),
*A. baumannii*		2.01 ± 1.84			3.31 (Kowalski, 2010), 0.71 (Templeton et al., 2009)
*P. aeruginosa*	1.99 ± 0.67	1.34 ± 0.79	1.49	2.21 (Clauss, 2006), 1.99 (Clauss et al., 2009), 1.51 (Narita et al., 2020), 1.97 (Clauss, 2006), 3.30 (Lakretz et al., 2010)	1.34 (McKinney and Pruden, 2012), 1.47 (Blatchley et al., 2017), 0.83 (Clauss, 2006), 0.75 (Clauss et al., 2009), 0.77 (Clauss, 2006), 2.27 (Lakretz et al., 2010), 2.75 (Rattanakul and Oguma, 2018),
*E. coli*	2.59 ± 3.09	3.42 ± 1.50	0.76	9.73^a^ (Gurzadyan et al., 1995), 2.11 (Clauss et al., 2009), 8.33 (Yin et al., 2015), 4.82 (Clauß et al., 2005), 1.69 (Narita et al., 2020), 2.53 (Matafonova et al., 2008), 2.65 (Clauß et al., 2005), 2.48 (Raeiszadeh and Taghipour, 2021)	3.05 (Guo et al., 2009), 3.20 (Chang et al., 1985), 0.97 (Clauss et al., 2009), 5.12 (Gurzadyan et al., 1995), 2.53 (Harris et al., 1987), 3.44 (Sommer et al., 1998), 3.51 (Sommer et al., 2000), 3.92 (Zimmer and Slawson, 2002), 3.40 (Clauß et al., 2005), 4.68 (Quek and Hu, 2008), 1.07 (Quek and Hu, 2008), 6.40 (Quek and Hu, 2008), 2.82 (Quek and Hu 2008), 5.90 (Quek and Hu, 2008), 4.43 (Sommer et al., 2000), 3.52 (Sommer et al., 2000), 2.47 (Sommer et al., 2000), 0.41 (Sommer et al., 2000), 5.00 (Sommer et al., 2000), 2.59 (Sommer et al., 2000), 4.02 (Sommer et al., 2000), 3.19 (Guo et al., 2009)
			(Average 1.48)		

a Irradiated at 216 nm. The errors derive from standard deviation.

The median log reduction dose for inactivation of *E. faecalis* was 8.36 ± 1.09 mJ/cm^2^ at 222 nm and is therefore 2.28-fold higher than the dose at 254 nm. The lowest difference between the median log reduction doses at both wavelength was determined for *E. coli*. For *P. aeruginosa*, the ratio of median log reduction doses at 222 and 254 nm are similar. No ratio could be determined for *A. baumannii* because of lack of literature data. The required median log reduction dose for *E. coli* is lower at 222 nm than at 254 nm. If the photoinactivation was compared on average for all pathogens at both wavelength, a 1.89-fold higher median log reduction dose was required at 222 nm than for a reduction at 254 nm.

**Figure 3:**
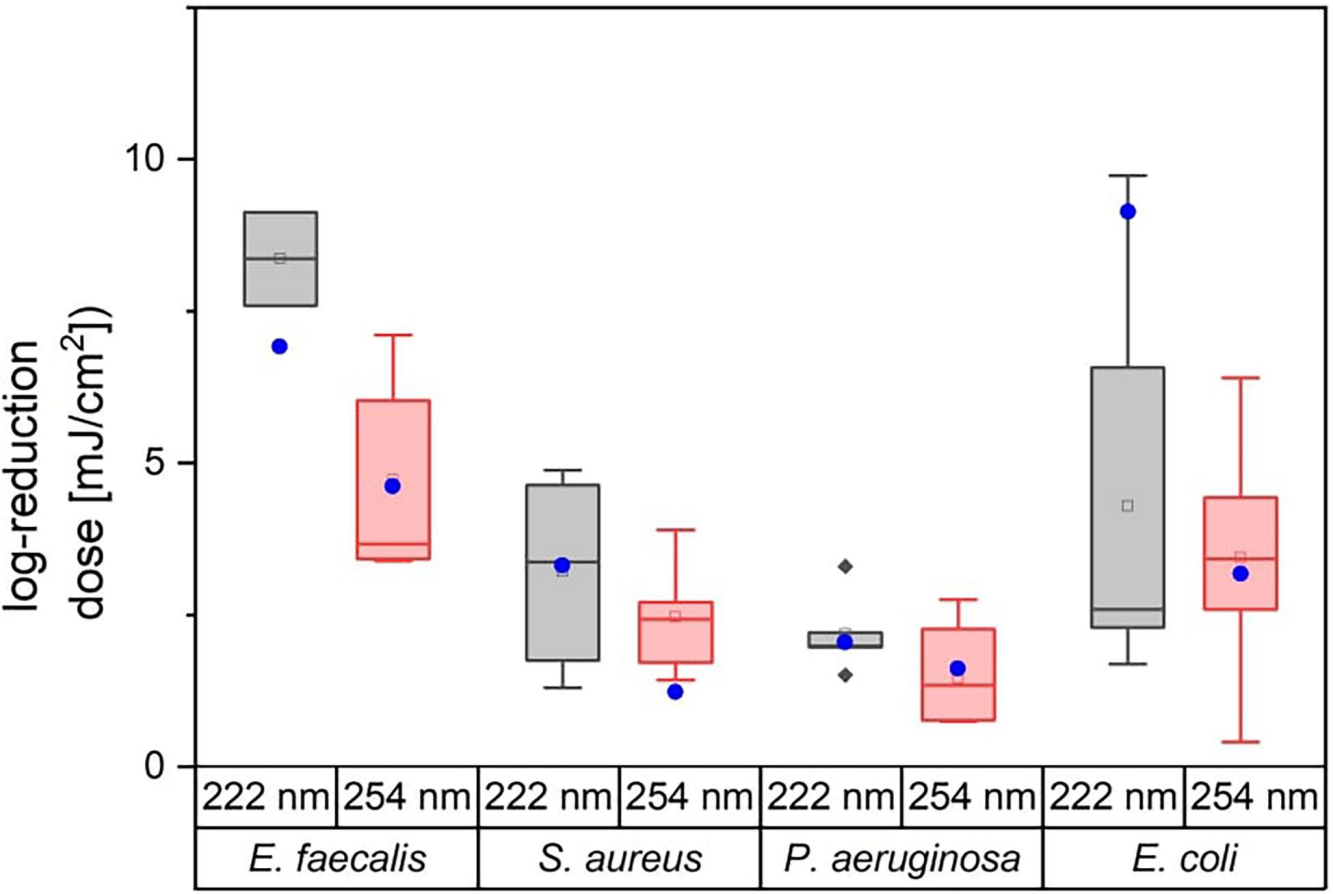
Overview of literature values for average log reduction doses of *E. faecalis*, *S. aureus*, *P. aeruginosa*, and *E. coli*. The attached blue circular dots represent the average log reduction doses of the investigated non-pathogenic relative. The corresponding data can be found in Table 2.

Figure 3 illustrates the variation of the literature values of ESKAPE pathogens. The colored boxes were created with the 25 and 75% quantiles from the median, whose cut-off was set as the threshold for surrogates. Five of eight measured doses of possible surrogates were within this range, with the exception of *E. mundtii*, non-pathogenic *E. coli* at 222 nm and *S. carnosus* at 254 nm. The deviations for *E. faecalis* were not clearly represented because of the small amount of data. The remaining average log reduction doses of the non-pathogenic relatives were within the 25 and 75% quantiles and were close to the median or mean log reduction doses of the ESKAPE pathogens. For the pathogenic *E. coli*, a larger scatter of values is observed for both wavelengths. At 222 nm, the median is further away from the average value. Compared to the other microorganisms, the enterococci required in contrast to pseudomonads higher average log reduction doses at both wavelengths.

In the following, the curve progression for both wavelengths are discussed and compared with literature values from other studies for pathogenic ESKAPE strains (Table 3). Members of the genus Staphylococcus, Enterococcus and Pseudomonas were inactivated with a lower dose at a wavelength of 254 nm. The lowest ratio between the non-pathogen relatives and the ESKAPE pathogens was determined for *P. fluorescens*, *P. aeruginosa* and *S. carnosus, S. aureus* at 222 nm. In contrast, the highest difference between both wavelengths with a 3.51-fold higher and a 0.52-fold lower reduction dose was ascertained for pathogenic, non-pathogenic *E. coli* at 222 nm and *S. carnosus, S. aureus* at 254 nm. Assuming that a threshold of 25% deviation of the ratio is set as limit of the log reduction dose of a non-pathogen relative to the pathogen, half of the values were within this limit (Table 3). The average log reduction doses for *E. mundtii* and *E. faecalis* exhibited a deviation of 17% at 222 nm and 24% at 254 nm. The ratios of *S. carnosus, S. aureus* at 254 nm, the ratios of *A. kookii, A. baumannii* at 254 nm and the ratios of non-pathogenic *E. coli* to pathogenic *E. coli* at 222 nm were above this assumed threshold percentage. It was also noticed that on average all pathogens need up to 1.48-fold higher irradiation dose at 222 nm than at 254 nm and compared to non-pathogen relatives, a 1.79-fold higher average dose was needed at 222 nm.

**Table 3:** Comparative overview of (average) log reduction doses for ESKAPE pathogens and non-pathogen relative.

222 nm				
Non-pathogenic bacteria	Average log reduction dose (mJ/cm^2^)	Pathogenic bacteria	Median log reduction dose (mJ/cm^2^)	Ratio (dose_222nm, non-pathogen_ /dose_222nm, pathogen_)
*E. mundtii*	6.90 ± 0.56	*E. faecalis*	8.36 ± 1.09	0.83
*S. carnosus*	3.28 ± 0.50	*S. aureus*	3.37 ± 1.48	0.97
*A. kookii*	2.93 ± 0.35	*A. baumannii*		
*P. fluorescens*	2.01 ± 0.14	*P. aeruginosa*	1.99 ± 0.67	1.01
*E. coli*	9.10 ± 2.11	*E. coli*	2.59 ± 3.09	3.51
254 nm				
	Average log reduction dose (mJ/cm^2^)		Median log reduction dose (mJ/cm^2^)	Ratio (dose_222nm, non-pathogen_ /dose_222nm, pathogen_)
*E. mundtii*	4.54 ± 0.34	*E. faecalis*	3.67 ± 1.73	1.24
*S. carnosus*	1.26 ± 0.06	*S. aureus*	2.43 ± 0.79	0.52
*A. kookii*	4.03 ± 0.74	*A. baumannii*	2.01 ± 1.84	2.00
*P. fluorescens*	1.59 ± 0.12	*P. aeruginosa*	1.34 ± 0.79	1.19
*E. coli*	3.19 ± 0.89	*E. coli*	3.42 ± 1.50	0.93

The errors derive from the standard deviation.

## Discussion

The annual proportion of illnesses caused by antimicrobial-resistant pathogens is increasing. Especially, the ESKAPE pathogens are particularly worthy of mention. To minimize possible spread, it is important to investigate further antimicrobial measures (Powers, 2004; Kowalski, 2010; Santajit et al., 2016). However, since working with such multidrug-resistant pathogens in laboratory is more difficult and often no laboratory with the necessary safety level is available, the search for suitable surrogates is important (Sinclair et al., 2012). For this reason, possible surrogates for photoinactivation experiments at 222 and 254 nm were investigated in this study. For this purpose, the measured data of the non-pathogenic relatives was compared with literature data of ESKAPE pathogens. For clarity of data and statements, the average and median log reduction doses were given with standard deviations and compared *via* a boxplot.

In the search of possible literature data of the reduction doses, many different methods and realizations have been noticed, with more data on experiments at 254 nm than at 222 nm (Malayeri et al., 2016; Hessling et al., 2021b; Masjoudi et al., 2021). However, since a similar performance has been desired to obtain clearer conclusions without unknown side effects, only data from studies with predefined criteria have been selected. There are also some studies that have investigated photoinactivation experiments for the food industry, using different media for irradiation, such as milk or even juices (Orlowska et al., 2015; Yin et al., 2015). When choosing such media, it must be taken into account that part of the emitted radiation is absorbed by the medium itself and therefore higher average log reduction doses are needed (Buonanno et al., 2017).

For both ESKAPE pathogens and non-pathogenic relatives, experiments at 222 nm require on average a higher log reduction dose (Tables 1, 2). Thus, irradiation at 254 nm is on average a more effective option for antimicrobial application. However, irradiation at this wavelength has been proven to be hazardous to health (Buonanno et al., 2017). The reason for this is the emission range, which is close to the absorption peak of DNA. When emitting at 222 nm, the emission range is also in the absorption range of proteins (Setlow and Doyle, 1957; Voet et al., 1963). Thus, an application with a radiation source emitting at 254 nm is more effective, but is not applicable to a patient due to the health hazard. However, the application can be used for irradiation of different clinical surfaces (Santos and Castro, 2021). Higher doses were determined on average at 222 nm for both the non-pathogenic relatives with 1.79 and ESKAPE pathogens with 1.48. Thus, a slightly higher irradiation dose would be required for direct human application, but with lower health risk. Despite these factors, there are bacteria among both non-pathogenic relatives and ESKAPE representatives that have a higher log reduction dose at 254 nm, such as *A. kookii* and pathogenic *E. coli*. Thus, in general, there is no clear tendency that ESKAPE pathogens are more sensitive at one wavelength than non-pathogen relatives. Furthermore, there is no correlation regarding photoinactivation by a wavelength and pathogenicity.

When comparing non-pathogenic relatives to ESKAPE pathogens *via* boxplot, it is noticeable that in five of eight cases, and thus in more than half of the cases, the investigated non-pathogenic relatives can be applied as surrogates. This has been studied for both wavelengths separately and it is noticeable that a bacterium might not generally be called a suitable surrogate, but it must be considered by wavelength. There are requirements whereby a surrogate can generally be determined (Sinclair et al., 2012). In the study of Sinclair et al. (2012), the aspects are not only in the photoinactivation but also in the taxonomy, genetics, partly also biochemistry or around resistances and also around inactivation. These are generally very important investigation criteria for possible surrogates. In addition, prioritization of surrogate attributes were explored to select possible surrogates and then use the appropriate surrogate to generate a public health risk assessment.

However, the focus of this study is on photoinactivation explicitly for the two wavelengths and here, clear differences can be observed. The possible surrogates are investigated statistically with the help of standard deviations and a comparison *via* boxplot with quantiles. It is noticed that in five of eight cases the measured data are within the range and thus, these bacteria are admissible as surrogates. However, there are larger deviations of reduction doses between non-pathogenic and pathogenic bacteria for *E. coli* at 222 nm and for *S. carnosus, S. aureus* at 254 nm. For *E. faecalis*, the statement regarding the surrogate property of Figure 3; Table 3 are contradictory, because a rate of log reduction doses of non-pathogen *E. mundtii* to pathogen *E. faecalis* with 17% can be determined, but the measured log reduction dose of *E. mundtii* is outside the quantile in the graph. The problem here is the paucity of underlying literature data. There is little data on *E. faecalis* especially at a wavelength of 222 nm. For statistical investigations, a larger data set should be deposited in order to make clear statements (Hu and Gurtler, 2017). Furthermore, it also reveals that the standard deviations usually differ a lot between the data of the non-pathogenic bacteria and the data from studies and thus, values close to the borderline can give a possibility of overlap. Since the experiments have been repeated several times in almost all studies, including here as triplicates, it is thus preferable to generally report log reduction doses with standard deviations. Based on the literature data for *E. coli*, it is also clear that the values of the reduction doses can vary despite selected criteria. *E. coli* is a frequently applied model organism in microbiology. Thus, many studies have also been found regarding photoinactivation. In one study, different *E. coli* strains are examined in relation to different light sources (Gurzadyan et al., 1995). It is reported that different strains are damaged differently by irradiation and thus, very different reduction doses can be calculated. So, different doses can be determined for different strains due to different repair mechanisms and gene expression of proteins, which also explains the differences in literature data on ESKAPE pathogens. Since the photoinactivation properties of the pathogens exhibit a certain scattering, the criterion for the determination of surrogates *via* a boxplot and the quantiles described therein is a reasonable method. It is determined that the log reduction dose of the surrogate is between the 25% quantile, where highest one quarter of the data is smaller than this dose, and the 75% quantile, where highest one quarter of the data is larger than this dose. Since potential surrogates, such as pathogens, also have different values in photoinactivation property due to various aspects, such as gene expression and others, the criterion regarding quantiles is a good way to see if the log reduction doses overlap despite the biology-related scatter.

The limitations of this study lie in the comparative studies on ESKAPE pathogens. Due to the legal restrictions on culturing of pathogens in the available laboratory, published studies on ESKAPE pathogens had to be applied for comparison. Accordingly, these trials were not conducted exactly the same and with the same setup as our experiments with the non-pathogenic surrogate candidates. Furthermore, the comparison is based on the determined doses for a 1 log reduction under the assumption of a strictly exponential behavior.

A further point is that not for all representatives of the ESKAPE pathogens statements could be received. For Klebsiella, no non-pathogenic representative was available worldwide, and no suitable studies for comparison could be found for *E. faecium* or *A. baumannii*, respectively.

## Conclusion

When working with pathogenic bacteria, especially multi-resistant bacteria, higher safety standards must be followed. So, the search for possible surrogates is an important issue. There are already specifications for which criteria a bacterium can generally be called a surrogate but it became apparent through the experiments that a surrogate cannot automatically assume this function for all experiments. To investigate this aspect for UVC photoinactivation, the literature values of log reduction doses were compared to the measured ones of non-pathogenic relatives *via* boxplot. It was assumed that a bacterium is a suitable surrogate at one wavelength if the average log reduction dose is within the quantile of the literature values. This is the case for nearly half of the investigated bacteria and wavelengths, except the values of *S. carnosus* below the 25% quantile at 254 nm and *E. coli* above the 75% quantile at 254 nm. No statement can be obtained for *A. kookii* due to insufficient data. The dose for *E. mundtii* at 222 nm is below the 25% quantile, although the data set is too small for a clear statement. Furthermore, the data should be presented with standard deviations or other statistical statements, as there will always be scatter in the log reduction doses due to various aspects, such as the gene expression of proteins of different bacteria of a strain, temperature, medium and others. Due to this scatter, the comparison of possible surrogates to pathogenic bacteria over a boxplot is very useful. However, in order to present the statements even more clearly, a larger data set of pathogenic bacteria is important and should be expanded. Since for some bacteria and wavelengths, the chosen criterion for surrogates was not met, other non-pathogenic relatives of ESKAPE pathogens should be investigated.

## Data Availability Statement

The original contributions presented in the study are included in the article/Supplementary Material, further inquiries can be directed to the corresponding author.

## Author Contributions

A-MG and MH conceived and designed the study, were involved in the interpretation of the results, and revised the manuscript. A-MG performed the experiments, analyzed the data, and wrote the paper. All authors contributed to the article and approved the submitted version.

## Conflict of Interest

The authors declare that the research was conducted in the absence of any commercial or financial relationships that could be construed as a potential conflict of interest.

## Publisher’s Note

All claims expressed in this article are solely those of the authors and do not necessarily represent those of their affiliated organizations, or those of the publisher, the editors and the reviewers. Any product that may be evaluated in this article, or claim that may be made by its manufacturer, is not guaranteed or endorsed by the publisher.

## Supplementary Material

The Supplementary Material for this article can be found online at: https://www.frontiersin.org/articles/10.3389/fmicb.2022.942708/full#supplementary-material

## References

[ref1] Acuff J. C. Wu J. Marik C. Waterman K. Gallagher D. Huang H.. (2020). Thermal inactivation of Salmonella, Shiga toxin-producing *Escherichia coli*, listeria monocytogenes, and a surrogate (*Pediococcus acidilactici*) on raisins, apricot halves, and macadamia nuts using vacuum-steam pasteurization. Int. J. Food Microbiol. 333:108814. doi: 10.1016/j.ijfoodmicro.2020.108814, PMID: 32805576

[ref2] Ahmed W. Bertsch P. M. Bibby K. Haramoto E. Hewitt J. Huygens F.. (2020). Decay of SARS-CoV-2 and surrogate murine hepatitis virus RNA in untreated wastewater to inform application in wastewater-based epidemiology. Environ. Res. 191:110092. doi: 10.1016/j.envres.2020.110092, PMID: 32861728 PMC7451058

[ref3] Baker C. A. Almeida G. Lee J. A. Gibson K. E. (2021). Pathogen and surrogate survival in relation to fecal Indicator Bacteria in freshwater Mesocosms. Appl. Environ. Microbiol. 87:e0055821. doi: 10.1128/AEM.00558-21, PMID: 34047635 PMC8276815

[ref4] Blatchley E. R. Oguma K. Sommer R. (2017). Comment on ‘UV disinfection induces a VBNC state in Escherichia coli and *Pseudomonas aeruginosa*’. IUVA News 18.3, 12–16.

[ref5] Buonanno M. Ponnaiya B. Welch D. Stanislauskas M. Randers-Pehrson G. Smilenov L.. (2017). Germicidal efficacy and mammalian skin safety of 222-nm UV light. Radiat. Res. 187, 493–501. doi: 10.1667/RR0010CC.1, PMID: 28225654 PMC5552051

[ref6] Burnham J. P. Olsen M. A. Kollef M. H. (2019). Re-estimating annual deaths due to multidrug-resistant organism infections. Infect. Control Hosp. Epidemiol. 40, 112–113. doi: 10.1017/ice.2018.304, PMID: 30463634 PMC6602528

[ref7] Casanova L. M. Waka B. (2013). Survival of a surrogate virus on N95 respirator material. Infect. Control Hosp. Epidemiol. 34, 1334–1335. doi: 10.1086/673994, PMID: 24225628

[ref8] Chang J. C. Ossoff F. Lobe D. C. Dorfman M. H. Dumais C. M. Qualls R. G.. (1985). UV inactivation of pathogenic and indicator microorganisms. Appl. Environ. Microbiol. 49, 1361–1365. doi: 10.1128/aem.49.6.1361-1365.1985, PMID: 2990336 PMC241729

[ref9] Chen P.-Y. Chu X.-N. Liu L. Hu J.-Y. (2016). Effects of salinity and temperature on inactivation and repair potential of *Enterococcus faecalis* following medium- and low-pressure ultraviolet irradiation. J. Appl. Microbiol. 120, 816–825. doi: 10.1111/jam.13026, PMID: 26669257

[ref10] Clauss M. (2006). Higher effectiveness of photoinactivation of bacterial spores, UV resistant vegetative bacteria and mold spores with 222 nm compared to 254 nm wavelength. Acta Hydrochim. Hydrobiol. 34, 525–532. doi: 10.1002/aheh.200600650

[ref11] Clauß M. Mannesmann R. Kolch A. (2005). Photoreactivation of *Escherichia coli* and *Yersinia enterolytica* after irradiation with a 222 nm Excimer lamp compared to a 254 nm low-pressure mercury lamp. Acta Hydrochim. Hydrobiol. 33, 579–584. doi: 10.1002/aheh.200400600

[ref12] Clauss M. Springorum A. C. Hartung J. (2009). Ultraviolet disinfection with 222 nm wavelength—new options to inactivate UV-resistant pathogens. in *Sustainable Animal Husbandry: Prevention is Better Than Cure 2*; July 19–23. Vechta, Germany.

[ref13] de Macedo V. Rodrigues G. H. Costa G. D. E. Oliveira E. R. Damasceno G. V. Mendonça J. S. P.. (2021). Interplay between ESKAPE Pathogens and Immunity in Skin Infections: An Overview of the Major Determinants of Virulence and Antibiotic Resistance. Pathogens 10:148. doi: 10.3390/pathogens10020148 33540588 PMC7912840

[ref14] Della-Porta T. (2008). Laboratory accidents and breaches in biosafety—they do occur! Microbiol. Aust. 29:62. doi: 10.1071/MA08062

[ref15] Eadie E. Barnard I. M. R. Ibbotson S. H. Wood K. (2021). Extreme exposure to filtered far-UVC: a case study†. Photochem. Photobiol. 97, 527–531. doi: 10.1111/php.13385, PMID: 33471372 PMC8638665

[ref16] Gates F. L. (1930). A study of the bactericidal action of ultra VIOLET light: iii. The absorption of ultra VIOLET light by BACTERIA. J. Gen. Physiol. 14, 31–42. doi: 10.1085/jgp.14.1.31, PMID: 19872573 PMC2141090

[ref17] Griffiths P. A. Babb J. R. Fraise A. P. (1998). *Mycobacterium terrae*: a potential surrogate for *Mycobacterium tuberculosis* in a standard disinfectant test. J. Hosp. Infect. 38, 183–192. doi: 10.1016/S0195-6701(98)90273-0, PMID: 9561469

[ref18] Guo M. Hu H. Bolton J. R. El-Din M. G. (2009). Comparison of low- and medium-pressure ultraviolet lamps: photoreactivation of *Escherichia coli* and total coliforms in secondary effluents of municipal wastewater treatment plants. Water Res. 43, 815–821. doi: 10.1016/j.watres.2008.11.028, PMID: 19081599

[ref19] Gurtler J. B. Rivera R. B. Zhang H. Q. Geveke D. J. (2010). Selection of surrogate bacteria in place of *E. coli* O157:H7 and *Salmonella Typhimurium* for pulsed electric field treatment of orange juice. Int. J. Food Microbiol. 139, 1–8. doi: 10.1016/j.ijfoodmicro.2010.02.023, PMID: 20223544

[ref20] Gurzadyan G. G. Görner H. Schulte-Frohlinde D. Gorner H. (1995). Ultraviolet (193, 216 and 254 nm) photoinactivation of *Escherichia coli* strains with different repair deficiencies. Radiat. Res. 141, 244–251. doi: 10.2307/3579001, PMID: 7871151

[ref21] Harm W. (1980). Biological Effects of Ultraviolet Radiation. Cambridge: Cambridge University Press (IUPAB Biophysics Series).

[ref22] Harris G. D. Adams V. D. Sorensen D. L. Curtis M. (1987). Ultraviolet inactivation of selected bacteria and viruses with photoreactivation of the bacteria. Water Res. 21, 687–692. doi: 10.1016/0043-1354(87)90080-7

[ref23] Hessling M. Haag R. Sicks B. (2021a). Review of microbial touchscreen contamination for the determination of reasonable ultraviolet disinfection dose. GMS Hyg. Infect. Control 16:Doc30. doi: 10.3205/dgkh000401 34956822 PMC8662742

[ref24] Hessling M. Haag R. Sieber N. Vatter P. (2021b). The impact of far-UVC radiation (200–230 nm) on pathogens, cells, skin, and eyes – a collection and analysis of a hundred years of data. GMS Hyg. Infect. Control 16:Doc07. doi: 10.3205/DGKH000378 33643774 PMC7894148

[ref25] Hu M. Gurtler J. B. (2017). Selection of surrogate Bacteria for use in food safety challenge studies: a review. J. Food Prot. 80, 1506–1536. doi: 10.4315/0362-028X.JFP-16-536, PMID: 28805457

[ref26] Hulkower R. L. Casanova L. M. Rutala W. A. Weber D. J. Sobsey M. D. (2011). Inactivation of surrogate coronaviruses on hard surfaces by health care germicides. Am. J. Infect. Control 39, 401–407. doi: 10.1016/j.ajic.2010.08.011, PMID: 21256627 PMC7132663

[ref27] Hung K.-F. Sidorova J. M. Nghiem P. Kawasumi M. (2020). The 6-4 photoproduct is the trigger of UV-induced replication blockage and ATR activation. Proc. Natl. Acad. Sci. U. S. A. 117, 12806–12816. doi: 10.1073/pnas.1917196117, PMID: 32444488 PMC7293618

[ref28] Ingham S. C. Algino R. J. Ingham B. H. Schell R. F. (2010). Identification of *Escherichia coli* O157:H7 surrogate organisms to evaluate beef carcass intervention treatment efficacy. J. Food Prot. 73, 1864–1874. doi: 10.4315/0362-028x-73.10.1864, PMID: 21067675

[ref29] Kang J.-W. Kim S.-S. Kang D.-H. (2018). Inactivation dynamics of 222 nm krypton-chlorine excilamp irradiation on gram-positive and gram-negative foodborne pathogenic bacteria. Food ReInt. 109, 325–333. doi: 10.1016/j.foodres.2018.04.018, PMID: 29803456

[ref30] Knaus J. Vatter P. Hessling M. (2021). Development and verification of a test rig for inactivation of bacteria and (corona-) viruses by UVC air disinfection systems. Curr. Dir. Biomed. Eng. 7, 315–318. doi: 10.1515/cdbme-2021-2080

[ref31] Kopit L. M. Kim E. B. Siezen R. J. Harris L. J. Marco M. L. (2014). Safety of the surrogate microorganism *Enterococcus faecium* NRRL B-2354 for use in thermal process validation. Appl. Environ. Microbiol. 80, 1899–1909. doi: 10.1128/AEM.03859-13, PMID: 24413604 PMC3957640

[ref32] Kowalski W. (2010). Ultraviolet Germicidal Irradiation Handbook. UVGI for Air and Surface Disinfection. Berlin, Heidelberg: Springer Berlin Heidelberg.

[ref33] Lai A. C. K. Cheung A. C. T. Wong M. M. L. Li W. S. (2016). Evaluation of cold plasma inactivation efficacy against different airborne bacteria in ventilation duct flow. Build. Environ. 98, 39–46. doi: 10.1016/j.buildenv.2015.12.005

[ref34] Lakretz A. Ron E. Z. Mamane H. (2010). Biofouling control in water by various UVC wavelengths and doses. Biofouling 26, 257–267. doi: 10.1080/08927010903484154, PMID: 20024789

[ref35] Li Z. Xie J. Yang J. Liu S. Ding Z. Hao J.. (2021). Pathogenic characteristics and risk factors for ESKAPE pathogens infection in burn patients. Infect. Drug Resist. 14, 4727–4738. doi: 10.2147/IDR.S338627, PMID: 34795489 PMC8594746

[ref36] Liu S. Rojas R. V. Gray P. Zhu M.-J. Tang J. (2018). *Enterococcus faecium* as a Salmonella surrogate in the thermal processing of wheat flour: influence of water activity at high temperatures. Food Microbiol. 74, 92–99. doi: 10.1016/j.fm.2018.03.001, PMID: 29706342

[ref37] Ludwig-Begall L. F. Wielick C. Dams L. Nauwynck H. Demeuldre P.-F. Napp A.. (2020). The use of germicidal ultraviolet light, vaporized hydrogen peroxide and dry heat to decontaminate face masks and filtering respirators contaminated with a SARS-CoV-2 surrogate virus. J. Hosp. Infect. 106, 577–584. doi: 10.1016/j.jhin.2020.08.025, PMID: 32889029 PMC7462546

[ref38] Malayeri A. H. Mohseni M. Cairns B. Bolton J. R. Chevrefils G. Caron E.. (2016). Fluence (UV dose) required to achieve incremental log inactivation of bacteria, protozoa, viruses and algae. IUVA News. 18, 4–6.

[ref39] Mariita R. M. Davis J. H. Randive R. V. (2022). Illuminating human Norovirus: A perspective on disinfection of water and surfaces using UVC, Norovirus model organisms, and radiation safety considerations. Pathogens 11:226. doi: 10.3390/pathogens11020226, PMID: 35215169 PMC8879714

[ref40] Mariita R. M. Randive R. V. (2021). Disinfection of methicillin-resistant *Staphylococcus aureus*, vancomycin-resistant *Enterococcus faecium* and *Acinetobacter baumannii* using Klaran WD array system. Access Microbiol. 3:194. doi: 10.1099/acmi.0.000194, PMID: 34712901 PMC8549383

[ref41] Martiny H. Wlodavezyk K. Rüden H. (1988). Anwendung von UV-Strahlen zur Desinfektion von Wasser. II. Mitteilung: Mikrobiologische Untersuchungen in Oberflächenwasser. Zentralblatt fur Bakteriologie, Mikrobiologie und Hygiene. Ser. B. Umwelthygiene, Krankenhaushygiene, Arbeitshygiene, praventive Medizin 186, 344–359. 3140539

[ref42] Masjoudi M. Mohseni M. Bolton J. R. (2021). Sensitivity of Bacteria, Protozoa, viruses, and other microorganisms to ultraviolet radiation. J. Res. Natl. Inst. Stand. Technol. 126, 1–77. doi: 10.6028/jres.126.021 PMC11259122 39081635

[ref43] Matafonova G. G. Batoev V. B. Astakhova A. Gómez M. Christofi N. (2008). Efficiency of KrCl excilamp (222 nm) for inactivation of bacteria in suspension. Lett. Appl. Microbiol. 47, 508–513. doi: 10.1111/j.1472-765X.2008.02461.x, PMID: 19120918

[ref44] McKinney C. W. Pruden A. (2012). Ultraviolet disinfection of antibiotic resistant Bacteria and their antibiotic resistance genes in water and wastewater. Environ. Sci. Technol. 46, 13393–13400. doi: 10.1021/es303652q 23153396

[ref45] Moreno-Andrés J. Romero-Martínez L. Acevedo-Merino A. Nebot E. (2016). Determining disinfection efficiency on *E. faecalis* in saltwater by photolysis of H2O2: implications for ballast water treatment. Chem. Eng. J. 283, 1339–1348. doi: 10.1016/j.cej.2015.08.079

[ref46] Moreno-Andrés J. Romero-Martínez L. Acevedo-Merino A. Nebot E. (2017). UV-based technologies for marine water disinfection and the application to ballast water: does salinity interfere with disinfection processes? Sci. Total Environ. 581-582, 144–152. doi: 10.1016/j.scitotenv.2016.12.077, PMID: 28011021

[ref47] Murray C. J. L. Ikuta K. S. Sharara F. Swetschinski L. Aguilar R. Gray A.. (2022). Global burden of bacterial antimicrobial resistance in 2019: a systematic analysis. Lancet 399, 629–655. doi: 10.1016/S0140-6736(21)02724-0, PMID: 35065702 PMC8841637

[ref48] Nakonieczna J. Wozniak A. Pieranski M. Rapacka-Zdonczyk A. Ogonowska P. Grinholc M. (2019). Photoinactivation of ESKAPE pathogens: overview of novel therapeutic strategy. Future Med. Chem. 11, 443–461. doi: 10.4155/fmc-2018-0329, PMID: 30901231

[ref49] Narita K. Asano K. Naito K. Ohashi H. Sasaki M. Morimoto Y.. (2020). Ultraviolet C light with wavelength of 222 nm inactivates a wide spectrum of microbial pathogens. J. Hosp. Infect. 105, 459–467. doi: 10.1016/j.jhin.2020.03.030 32243946

[ref50] Nerandzic M. M. Cadnum J. L. Eckart K. E. Donskey C. J. (2012). Evaluation of a hand-held far-ultraviolet radiation device for decontamination of Clostridium difficile and other healthcare-associated pathogens. BMC Infect. Dis. 12, 1–6. doi: 10.1186/1471-2334-12-120, PMID: 22591268 PMC3419611

[ref51] Niebuhr E. Laury A. Acuff G. R. Dickson J. S. (2008). Evaluation of nonpathogenic surrogate bacteria as process validation indicators for *Salmonella enterica* for selected antimicrobial treatments, cold storage, and fermentation in meat. J. Food Prot. 71, 714–718. doi: 10.4315/0362-028x-71.4.714, PMID: 18468024

[ref52] Normile D. (2004). Infectious diseaseMounting lab accidents raise SARS fear. Science 304, 659–661. doi: 10.1126/science.304.5671.659 15118129

[ref53] Oliveira D. M. P. d. Forde B. M. Kidd T. J. Harris P. N. A. Schembri M. A. Beatson S. A.. (2020). Antimicrobial resistance in ESKAPE pathogens. Clin. Microbiol. Rev. 33:e00181-19. doi: 10.1128/CMR.00181-19, PMID: 32404435 PMC7227449

[ref54] Orlowska M. Koutchma T. Kostrzynska M. Tang J. (2015). Surrogate organisms for pathogenic O157:H7 and non-O157 *Escherichia coli* strains for apple juice treatments by UV-C light at three monochromatic wavelengths. Food Control 47, 647–655. doi: 10.1016/j.foodcont.2014.08.004

[ref55] Palmer K. L. Godfrey P. Griggs A. Kos V. N. Zucker J. Desjardins C.. (2012). Comparative genomics of enterococci: variation in *Enterococcus faecalis*, clade structure in *E. faecium*, and defining characteristics of *E. gallinarum* and *E. casseliflavus*. mBio 3, e00318–e00311. doi: 10.1128/mBio.00318-11, PMID: 22354958 PMC3374389

[ref56] Park S. Kim C.-H. Jeong S. T. Lee S. Y. (2018). Surrogate strains of human pathogens for field release. Bioengineered 9, 17–24. doi: 10.1080/21655979.2017.1349044, PMID: 28692329 PMC5972925

[ref57] Powers J. H. (2004). Antimicrobial drug development--the past, the present, and the future. Clin. Microbiol. Infect. 10, 23–31. doi: 10.1111/j.1465-0691.2004.1007.x 15522037

[ref58] Que Y. A. Haefliger J. A. Francioli P. Moreillon P. (2000). Expression of *Staphylococcus aureus* clumping factor A in *Lactococcus lactis* subsp. cremoris using a new shuttle vector. Infect. Immun. 68, 3516–3522. doi: 10.1128/IAI.68.6.3516-3522.2000, PMID: 10816506 PMC97637

[ref59] Quek P. H. Hu J. (2008). Indicators for photoreactivation and dark repair studies following ultraviolet disinfection. J. Ind. Microbiol. Biotechnol. 35, 533–541. doi: 10.1007/s10295-008-0314-0, PMID: 18228066

[ref60] Raeiszadeh M. Taghipour F. (2021). Inactivation of microorganisms by newly emerged microplasma UV lamps. Chem. Eng. J. 413:127490. doi: 10.1016/j.cej.2020.127490

[ref61] Rahmani B. Hafsa C. Rahmani E.-r. Kacem M. Harche M. K. Bhosle S.. (2010). Photoinactivation of the *Escherichia coli* by the pulsed dielectric barrier discharge Excilamp krypton chlorine emitted at 222 nm. IEEE Trans. Plasma Sci. 38, 953–956. doi: 10.1109/TPS.2010.2041471

[ref62] Rattanakul S. Oguma K. (2018). Inactivation kinetics and efficiencies of UV-LEDs against *Pseudomonas aeruginosa*, legionella pneumophila, and surrogate microorganisms. Water Res. 130, 31–37. doi: 10.1016/j.watres.2017.11.047, PMID: 29195159

[ref63] Santajit S. Indrawattana N. Tulkens P. M. (2016). Mechanisms of antimicrobial resistance in ESKAPE pathogens. Biomed. Res. Int. 2016:2475067, –2475068. doi: 10.1155/2016/2475067, PMID: 27274985 PMC4871955

[ref64] Santos T. D. Castro L. F. d. (2021). Evaluation of a portable ultraviolet C (UV-C) device for hospital surface decontamination. Photodiagn. Photodyn. Ther. 33:102161. doi: 10.1016/j.pdpdt.2020.102161, PMID: 33373741 PMC7764389

[ref65] Schirtzinger E. E. Kim Y. Davis A. S. (2022). Improving human coronavirus OC43 (HCoV-OC43) research comparability in studies using HCoV-OC43 as a surrogate for SARS-CoV-2. J. Virol. Methods 299:114317. doi: 10.1016/j.jviromet.2021.114317, PMID: 34634321 PMC8500843

[ref66] Serrano-Aroca Á. (2022). Antiviral characterization of advanced materials: use of bacteriophage phi 6 as surrogate of enveloped viruses such as SARS-CoV-2. Int. J. Mol. Sci. 23:5335. doi: 10.3390/ijms23105335, PMID: 35628148 PMC9141689

[ref67] Setlow R. Doyle B. (1957). The action of monochromatic ultaviolet light on proteins. Biochim. BiophyActa 24, 27–41. doi: 10.1016/0006-3002(57)90142-7, PMID: 13426198

[ref68] Sharp D. G. (1939). The lethal action of short ultraviolet rays on several common pathogenic Bacteria. J. Bacteriol. 37, 447–460. doi: 10.1128/jb.37.4.447-460.1939, PMID: 16560218 PMC374478

[ref69] Shrivastava S. R. B. L. Shrivastava P. S. Ramasamy J. (2018). World health organization releases global priority list of antibiotic-resistant bacteria to guide research, discovery, and development of new antibiotics. J. Med. Soc. 32:76. doi: 10.4103/jms.jms_25_17

[ref70] Sinclair R. G. Rose J. B. Hashsham S. A. Gerba C. P. Haas C. N. (2012). Criteria for selection of surrogates used to study the fate and control of pathogens in the environment. Appl. Environ. Microbiol. 78, 1969–1977. doi: 10.1128/AEM.06582-11, PMID: 22247166 PMC3298155

[ref71] Singh G. Jorgenson J. Pringle T. Nelson T. Ramamoorthy S. (2021). Monitoring SARS-CoV-2 decontamination by dry heat and ultraviolet treatment with a swine coronavirus as a surrogate. Infect. Prev. Pract. 3, 3:100103. doi: 10.1016/j.infpip.2020.100103, PMID: 34316570 PMC7694467

[ref72] Sommer R. Haider T. Cabaj A. Pribil W. Lhotsky M. (1998). Time dose reciprocity in UV disinfection of water. Water Sci. Technol. 38, 145–150. doi: 10.1016/S0273-1223(98)00816-6

[ref73] Sommer R. Lhotsky M. Haider T. Cabaj A. (2000). UV inactivation, liquid-holding recovery, and photoreactivation of *Escherichia coli* O157 and other pathogenic *Escherichia coli* strains in water. J. Food Prot. 63, 1015–1020. doi: 10.4315/0362-028x-63.8.1015, PMID: 10945573

[ref74] Sosnin E. A. Avdeev M. Kuznetzova E. A. Lavrent'eva L. V. (2005). A bactericidal barrier-discharge KrBr excilamp. Instrum. Exp. Tech. 48, 663–666. doi: 10.1007/s10786-005-0118-7

[ref75] String G. M. White M. R. Gute D. M. Mühlberger E. Lantagne D. S. (2021). Selection of a SARS-CoV-2 surrogate for use in surface disinfection efficacy studies with chlorine and antimicrobial surfaces. Environ. Sci. Technol. Lett. 8, 995–1001. doi: 10.1021/acs.estlett.1c00593 37566364

[ref76] Stutzmann Meier P. Entenza J. M. Vaudaux P. Francioli P. Glauser M. P. Moreillon P. (2001). Study of *Staphylococcus aureus* pathogenic genes by transfer and expression in the less virulent organism *Streptococcus gordonii*. Infect. Immun. 69, 657–664. doi: 10.1128/IAI.69.2.657-664.2001, PMID: 11159952 PMC97936

[ref77] Taylor W. Camilleri E. Craft D. L. Korza G. Granados M. R. Peterson J.. (2020). DNA damage kills bacterial spores and cells exposed to 222-nanometer UV radiation. Appl. Environ. Microbiol. 86:e03039-19. doi: 10.1128/AEM.03039-19, PMID: 32033948 PMC7117916

[ref78] Templeton M. R. Antonakaki M. Rogers M. (2009). UV Dose–Response of *Acinetobacter baumannii* in Water. Environ. Eng. Sci. 26, 697–701. doi: 10.1089/ees.2008.0048

[ref79] Voet D. Gratzer W. B. Cox R. A. Doty P. (1963). Absorption spectra of nucleotides, polynucleotides, and nucleic acids in the far ultraviolet. Biopolymers 1, 193–208. doi: 10.1002/bip.360010302

[ref80] Wang H. Wang J. Li S. Ding G. Wang K. Zhuang T.. (2020). Synergistic effect of UV/chlorine in bacterial inactivation, resistance gene removal, and gene conjugative transfer blocking. Water Res. 185:116290. doi: 10.1016/j.watres.2020.116290, PMID: 32818733

[ref81] Whitworth C. Mu Y. Houston H. Martinez-Smith M. Noble-Wang J. Coulliette-Salmond A.. (2020). Persistence of bacteriophage phi 6 on porous and nonporous surfaces and the potential for its use as an Ebola virus or coronavirus surrogate. Appl. Environ. Microbiol. 86:e01482-20. doi: 10.1128/AEM.01482-20, PMID: 32591388 PMC7440805

[ref82] Yang C. Sun W. Ao X. (2020). Bacterial inactivation, DNA damage, and faster ATP degradation induced by ultraviolet disinfection. Front. Environ. Sci. Eng. 14, 1–10. doi: 10.1007/s11783-019-1192-6

[ref83] Yin F. Zhu Y. Koutchma T. Gong J. (2015). Inactivation and potential reactivation of pathogenic *Escherichia coli* O157:H7 in bovine milk exposed to three monochromatic ultraviolet UVC lights. Food Microbiol. 49, 74–81. doi: 10.1016/j.fm.2015.01.014, PMID: 25846917

[ref84] Yun J. Yan R. Fan X. Gurtler J. Phillips J. (Eds.) (2013). Fate of *E. coli* O157:H7, Salmonella spp. and potential surrogate bacteria on apricot fruit, following exposure to UV-C light. Int. J. Food Microbiol. 166, 356–363. doi: 10.1016/j.ijfoodmicro.2013.07.021, PMID: 24021820

[ref85] Zimmer J. L. Slawson R. M. (2002). Potential repair of *Escherichia coli* DNA following exposure to UV radiation from both medium- and low-pressure UV sources used in drinking water treatment. Appl. Environ. Microbiol. 68, 3293–3299. doi: 10.1128/AEM.68.7.3293-3299.2002, PMID: 12089006 PMC126789

