# Supplementary material for: Investigation on Potential ESKAPE Surrogates for 222 and 254 nm Irradiation Experiments
Source: Front Microbiol. 2022 Jul 1;13:942708. doi: 10.3389/fmicb.2022.942708 (PMC9284107; doi:10.3389/fmicb.2022.942708)
Supplement: Supplementary file 1 [file Data_Sheet_1.pdf]

Supplementary Material

1 Supplementary Figures and Tables

1.1 Supplementary Figure

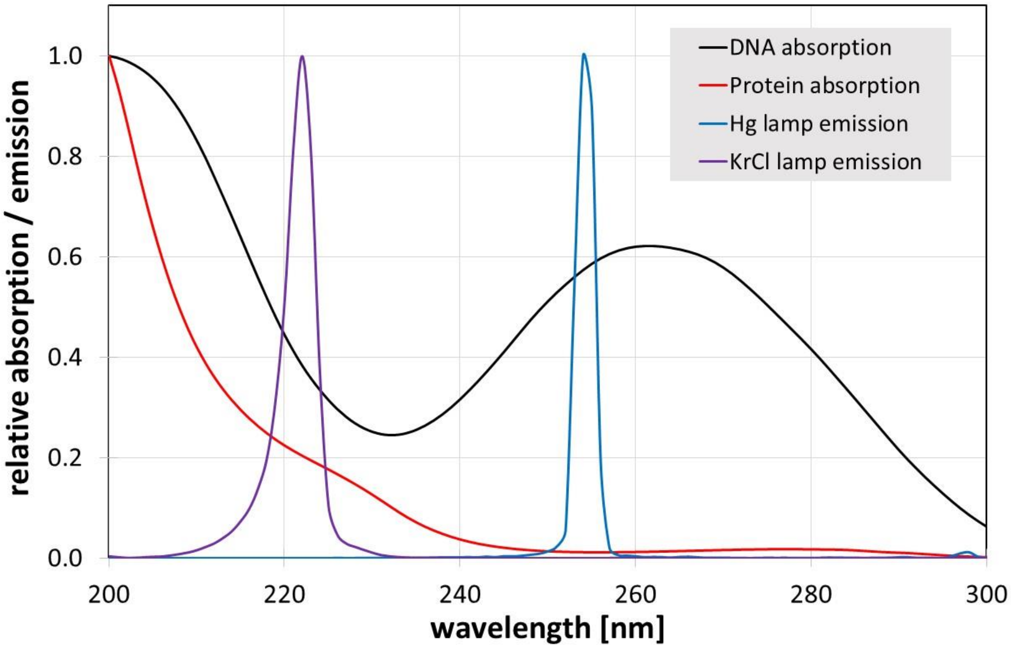

Supplementary Figure 1. Relative absorption spectra of DNA and proteins and emission spectra of a Hg and a filtered KrCl lamp (Representation according to (Hessling et al. 2021b)).

1.2 Supplementary Tables

Supplementary Table 1. Relative absorption spectra of DNA and proteins and emission spectra of Hg and filtered KrCl lamp (Representation according to (Hessling et al. 2021b)).

wavelength [nm]	DNA absorption	protein absorption	Hg lamp emission	KrCl lamp emission
200	1.000	1.000	0.000410	0.004040
201	0.996	0.940	0.000374	0.002315
202	0.990	0.870	0.000402	0.000978
203	0.981	0.796	0.000627	0.000881
204	0.970	0.724	0.000479	0.002263
205	0.955	0.658	0.000426	0.002976
206	0.938	0.599	0.000297	0.003596
207	0.917	0.545	0.000415	0.005624
208	0.892	0.498	0.000460	0.008414
209	0.863	0.456	0.000428	0.011648
210	0.831	0.420	0.000300	0.016152
211	0.796	0.388	0.000233	0.022637
212	0.758	0.360	0.000549	0.029859
213	0.719	0.336	0.000430	0.040702
214	0.679	0.314	0.000424	0.054060
215	0.638	0.295	0.000398	0.074058
216	0.597	0.278	0.000485	0.099599
217	0.556	0.262	0.000344	0.140601
218	0.517	0.249	0.000425	0.201502
219	0.479	0.236	0.000307	0.320004
220	0.444	0.224	0.000501	0.513676
221	0.412	0.214	0.000456	0.818179
222	0.383	0.204	0.000489	1.000000
223	0.357	0.195	0.000464	0.806768
224	0.335	0.186	0.000312	0.374527
225	0.314	0.177	0.000328	0.104008

226	0.296	0.167	0.000729	0.046952
227	0.281	0.158	0.000487	0.029576
228	0.268	0.148	0.000597	0.022839
229	0.258	0.137	0.000843	0.016771
230	0.251	0.126	0.000844	0.011139
231	0.246	0.115	0.000921	0.006402
232	0.244	0.103	0.000638	0.003904
233	0.245	0.092	0.000607	0.002547
234	0.249	0.081	0.000895	0.001571
235	0.255	0.071	0.000917	0.001120
236	0.263	0.063	0.001253	0.000762
237	0.274	0.055	0.001476	0.000646
238	0.286	0.048	0.002131	0.000546
239	0.300	0.043	0.001924	0.000336
240	0.316	0.037	0.002123	0.000342
241	0.333	0.033	0.002345	0.000372
242	0.352	0.029	0.001995	0.000353
243	0.371	0.026	0.003446	0.000327
244	0.390	0.023	0.002292	0.000402
245	0.411	0.021	0.004297	0.000431
246	0.432	0.019	0.003867	0.000452
247	0.453	0.017	0.005197	0.000445
248	0.474	0.016	0.007017	0.000416
249	0.493	0.015	0.009197	0.000422
250	0.511	0.014	0.013895	0.000391
251	0.529	0.013	0.023400	0.000355
252	0.544	0.013	0.054994	0.000293
253	0.560	0.012	0.538061	0.000265
254	0.574	0.012	1.000000	0.000346
255	0.586	0.012	0.895187	0.000354
256	0.597	0.012	0.193704	0.000427
257	0.605	0.012	0.020800	0.000532

258	0.612	0.012	0.006360	0.000539
259	0.617	0.012	0.005304	0.000425
260	0.620	0.013	0.004235	0.000261
261	0.621	0.013	0.002309	0.000235
262	0.621	0.013	0.002704	0.000207
263	0.620	0.013	0.002432	0.000181
264	0.618	0.014	0.001622	0.000143
265	0.614	0.014	0.002797	0.000114
266	0.610	0.015	0.003145	0.000089
267	0.605	0.015	0.002231	0.000113
268	0.598	0.016	0.001043	0.000139
269	0.589	0.016	0.001106	0.000138
270	0.578	0.016	0.001266	0.000114
271	0.565	0.017	0.000876	0.000141
272	0.551	0.017	0.000857	0.000122
273	0.536	0.017	0.000421	0.000157
274	0.520	0.018	0.000726	0.000284
275	0.503	0.018	0.001230	0.000362
276	0.486	0.018	0.000888	0.000382
277	0.468	0.018	0.001093	0.000407
278	0.451	0.018	0.000647	0.000447
279	0.433	0.018	0.000795	0.000450
280	0.415	0.018	0.000517	0.000497
281	0.396	0.018	0.000838	0.000547
282	0.377	0.017	0.001488	0.000583
283	0.357	0.017	0.001159	0.000584
284	0.336	0.016	0.000870	0.000634
285	0.316	0.016	0.000809	0.000627
286	0.296	0.015	0.000393	0.000690
287	0.275	0.014	0.000638	0.000716
288	0.255	0.013	0.000614	0.000674
289	0.234	0.012	0.001144	0.000701

290	0.214	0.011	0.002363	0.000689
291	0.195	0.010	0.002288	0.000592
292	0.177	0.009	0.000963	0.000608
293	0.160	0.008	0.000867	0.000644
294	0.144	0.007	0.000556	0.000575
295	0.128	0.006	0.000734	0.000530
296	0.113	0.005	0.004385	0.000553
297	0.098	0.004	0.010086	0.000598
298	0.085	0.003	0.012063	0.000572
299	0.073	0.003	0.003014	0.000529
300	0.062	0.002	0.001086	0.000517

Supplementary Table 2. Overview of the studied bacterial strains and the used media for this purpose.

**Strain**	**Temperature**	**Medium**	**Ingredients per 1000 ml**
E. moraviensis,<br>*S. carnosus*	37 °C	M92	30 g tryptic soy broth,<br>3 g yeast extract
A. kookii	37 °C	M220	15 g peptone from casein,<br>5 g peptone from soymeal,<br>5 g sodium chloride
P. fluorescens	30 °C	M535	5 g peptone from casein,<br>3 g meat extract
E. coli	37 °C	LB	10 g tryptone,<br>5 g yeast extract,<br>10 g sodium chloride
